# Normalised Diagnostic Contribution Index (NDCI) Integration to Multi Objective Sensor Optimisation Framework (MOSOF)—An Environmental Control System Case

**DOI:** 10.3390/s25092661

**Published:** 2025-04-23

**Authors:** Burak Suslu, Fakhre Ali, Ian K. Jennions

**Affiliations:** Integrated Vehicle Health Management Centre, School of Aerospace, Transport and Manufacturing, Cranfield University, Cranfield MK43 0AL, UK; f.ali@cranfield.ac.uk (F.A.); i.jennions@cranfield.ac.uk (I.K.J.)

**Keywords:** multi-objective sensor optimisation, normalised diagnostic contribution index, aircraft, environmental control systems, diagnostics, complex systems, health management

## Abstract

In modern aerospace systems, effective sensor optimisation is essential for ensuring reliable diagnostics, efficient resource allocation, and proactive maintenance. This paper presents Normalised Diagnostic Contribution Index (NDCI) integration into the Multi-Objective Sensor Optimisation Framework (MOSOF) to address application-specific performance nuances. Building on previous work, the proposed approach leverages a multi-objective genetic algorithm to optimise key criteria, including performance, cost, reliability management, and compatibility. NDCI is derived from simulation data obtained via the Boeing 737-800 Environmental Control System (ECS) using the SESAC platform, where degradation level readings across four fault modes are analysed. The framework evaluates sensor performance from the perspectives of Original Equipment Manufacturers (OEM), Airlines, and Maintenance Repair Overhaul (MRO) organisations. Validation against the Minimum Redundancy Maximum Relevance (mRMR) method highlights the distinct advantage of NDCI by identifying an optimal set of three sensors compared to mRMR’s six-sensor solution, and MOSOF’s multi-objective insertion enhances sensor deployment for different stakeholders. This integration not only expands the feasible solution space for sensor-pair configurations but also emphasises diagnostic value over redundancy. Overall, the enhanced NDCI-MOSOF offers a scalable, multi-stakeholder approach for next-generation sensor optimisation and predictive maintenance in complex aerospace systems. The results demonstrate significant improvements in diagnostics efficiency for stakeholders.

## 1. Introduction

Modern aerospace and power generation systems increasingly depend on sophisticated sensor networks to ensure reliable fault detection, rapid isolation, and timely maintenance actions [1]. Advances in sensor technology have enabled the deployment of extensive sensor suites across a wide array of applications—from industrial process control and environmental monitoring to security and safety-critical operations [1,2]. In these complex systems, overall performance hinges on the careful selection and configuration of sensors—a process that must balance conflicting objectives, such as diagnostic accuracy, cost efficiency, reliability, and integration complexity. For instance, while original equipment manufacturers (OEMs) emphasise design simplicity and certification compliance, airline operators focus on minimising operational disruptions and maintenance expenses, and Maintenance, Repair, and Overhaul (MRO) providers prioritise swift fault isolation [2].

**Figure 1:**
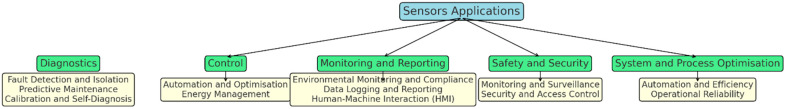
Sensor application domains.

Figure 1 illustrates the diverse domains where sensors play a pivotal role, including diagnostics, control, monitoring, safety, and system optimisation. Diagnostic applications benefit from robust fault detection and predictive maintenance schedules; control systems require real-time adjustments to optimise performance; monitoring tasks ensure environmental compliance and accurate data logging; and safety systems are essential for protecting assets and personnel.

Traditional sensor selection strategies have typically focused on either maximising diagnostic accuracy through high redundancy or reducing sensor counts to minimise costs. However, approaches that concentrate solely on one objective often prove suboptimal. High-redundancy configurations can drive up expenses without corresponding improvements in fault detection, while overly cost-driven designs risk undermining coverage of critical components [2]. Techniques such as the Minimum Redundancy Maximum Relevance (mRMR) algorithm have been widely employed to balance high relevance with low redundancy, thereby identifying sensor signals that are both informative and non-overlapping [3]. Recent enhancements to mRMR—including energy optimisation for wireless sensor networks [4] and improved measures of redundancy and relevance [5]—further refine its performance in feature selection and classification tasks. Furthermore, several design optimisation frameworks have been proposed to integrate testability and maintainability requirements into sensor selection processes, as illustrated by works on optimal sensor selection for health monitoring systems [6] and aerospace system health management under uncertainty [7].

Recent investigations into aircraft Environmental Control Systems (ECS) have highlighted these challenges. Legacy ECS designs—exemplified by early Boeing 737 models that employed only a couple of temperature sensors for control [8]—often struggle to detect subtle faults, such as heat exchanger fouling or valve leakage. Empirical studies have demonstrated that sensor selection methods based solely on traditional criteria may underperform in the presence of multiple fault modes and varying operational conditions, as shown in cross-condition fault diagnosis studies [9]. In response, researchers have developed advanced multi-objective optimisation techniques that integrate comprehensive diagnostic metrics with practical constraints. For example, integrated combinatorial approaches employing reduced order models and Gaussian processes have been proposed to further enhance sensor placement optimisation [10].

Central to these advanced methodologies is the Normalised Diagnostic Contribution Index (NDCI), which consolidates critical attributes—such as sensitivity, fault-separation capability, and uniqueness—into a single quantitative measure of diagnostic performance [11,12]. When integrated within the Multi-Objective Sensor Optimisation Framework (MOSOF) [13], the NDCI enables a holistic evaluation of each sensor’s contribution to fault detection, thereby facilitating a balanced assessment that accounts for both technical performance and resource constraints. In parallel, PHM-oriented sensor optimisation models, such as those applied to aircraft engines, have shown promising results in aligning sensor selection with prognostics and health management objectives [14]. Advanced fault diagnosis techniques—like subspace approaches to multidimensional fault identification and reconstruction—complement these frameworks by enhancing the capability to detect and isolate faults across multiple dimensions [15]. The exclusion of the structural health management applications due to high number of the studies presented in the literature was mentioned previously in the framework paper [13].

Building on these developments, this work embeds the NDCI within MOSOF and employs a Multi-Objective Genetic Algorithm (MOGA) to refine the sensor selection process for complex diagnostic applications. This enhanced framework simultaneously optimises four critical objectives:

- Overall Performance: A composite index that incorporates the normalised NDCI alongside other performance measures.
- Cost of Sensor Deployment: Minimising expenses while ensuring adequate diagnostic coverage.
- Reliability and Operational Efficiency: Enhancing system robustness through optimal sensor integration.
- Secondary Criteria: Considerations such as OEM compatibility or benefit-to-cost ratios, particularly pertinent to airline operations and MRO scenarios [16].

In practice, while traditional methods like mRMR may suggest larger sensor sets to guarantee redundancy, the NDCI-based approach has demonstrated the ability to achieve comparable fault detection with fewer sensors, thereby enhancing overall diagnostic efficiency. The optimisation process addresses the NP-complete nature of sensor placement [13] by initially employing mRMR as a pre-processing step to eliminate redundant signals and subsequently applying MOGA to navigate the complex combinatorial search space, thereby deriving Pareto-optimal solutions that balance multiple objectives [17]. System-level fault diagnosis studies applied to aircraft ECS further reinforce the value of such integrated optimisation approaches [18]. Moreover, multi-objective sensor placement strategies have been explored for composite aircraft structures using Kriging-based approaches, which provide valuable insights into optimal sensor layouts in complex material systems [19].

Case studies further validate the efficacy of this integrated approach. For instance, simulation studies on a Boeing 737-800 ECS have demonstrated that augmenting legacy configurations with strategically selected sensors—guided by the NDCI—can substantially improve fault detection and isolation capabilities [20]. Similarly, multi-objective optimisation strategies have been successfully applied to turbofan engine monitoring, where an optimised sensor set reduced 35 variables to just four critical indicators without compromising diagnostic accuracy [21]. Other domains, such as helicopter drive systems and polymer electrolyte membrane fuel cell prognostics, have also benefited from these methodologies [22,23]. Additionally, innovations like Teledyne’s Aircraft Cabin Environment Sensor (ACES) for the Boeing 737 exemplify the trend toward comprehensive sensor optimisation, balancing extensive environmental monitoring with practical constraints [20]. Design-for-diagnosability practices in modern systems, such as those implemented in the Boeing 787′s electric ECS, further underscore the importance of rigorous multi-objective trade studies in justifying sensor additions with respect to weight and power consumption [24,25].

Moreover, intellectual property considerations highlight the competitive advantages of optimised sensor layouts, with several patents underscoring the value of these innovations [26,27]. In summary, the evolution from traditional, single-objective sensor selection to sophisticated multi-objective frameworks like MOSOF represents a significant advancement in the design and operation of complex engineering systems. By integrating diagnostic metrics such as the NDCI with multi-objective optimisation algorithms—and by leveraging emerging techniques in sensor network design and fault diagnosis—the approach not only expands the feasible solution space for sensor configurations but also meets the diverse requirements of OEMs, Airlines, and MRO stakeholders. This work enhances fault detection and system reliability while laying the foundation for future research aimed at refining multi-stakeholder decision-making in sensor network design.

The key contributions of this work are listed below:

- Integration of Non-Dominated Criteria Identification (NDCI) into the Multi-Objective Sensor Optimisation Framework (MOSOF) for enhanced diagnostics applications.
- Application in Boeing 737-800 ECS Simulation (SESAC), focusing on degradation modes.
- Optimal resource utilisation and sensor design implementation aspects.
- Scalable framework addressing multi-stakeholder requirements in complex systems.

The remainder of this paper is organised as follows: Section 2 presents the theoretical underpinnings and methodology for integrating NDCI into MOSOF, including detailed explanations of sensor metrics and data interpretation. Section 3 formulates the multi-objective optimisation problem and describes the solution strategy using evolutionary algorithms. Section 4 discusses simulation results, comparative analyses, and presents improved figures. Section 5 offers a comprehensive discussion on practical implementation, scalability, and potential limitations. Finally, Section 6 concludes the study with remarks on future research directions.

## 2. NDCI Integration into MOSOF

### 2.1. Rationale for NDCI-Centric Sensor Evaluation

MOSOF was developed as a general approach to sensor implementation in complex systems, addressing multiple performance, cost, and reliability criteria. However, MOSOF’s broad scope sometimes masks the specific performance nuances essential for applications requiring precise sensor differentiation. The NDCI has emerged due to this work as a robust metric that quantifies sensor performance by evaluating parameters such as signal separation, sensitivity, and uniqueness. Integrating NDCI into MOSOF refines the sensor evaluation process by introducing an objective, quantitative measure of sensor quality that is particularly beneficial in applications where subtle performance differences can significantly impact system outcomes.

**Figure 2:**
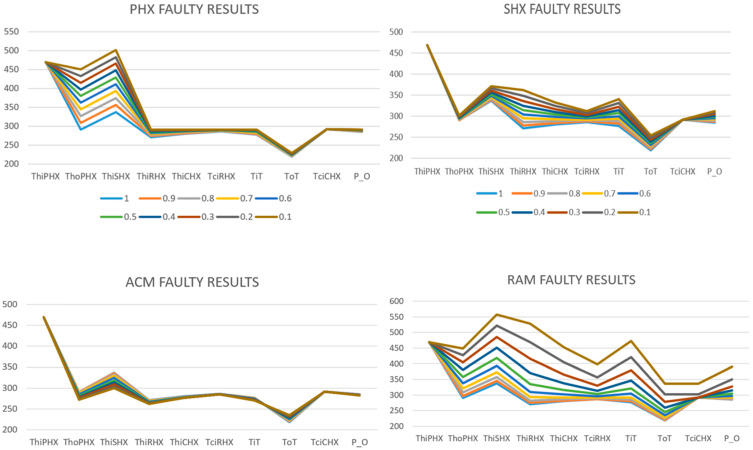
Temperature sensors’ readings across ECS components obtained from SESAC simulation for four fault modes [28].

The temperature profiles shown in Figure 2 highlight distinct variations in sensor readings across the ECS components when subjected to fault conditions. Notably, each fault scenario—Primary Heat Exchanger (PHX), Secondary Heat Exchanger (SHX), Air-Cycle-Machine (ACM), and Ram Air—generates unique patterns of temperature deviations that facilitate fault identification and isolation. For instance, PHX faults predominantly elevate temperature readings at the initial stages due to reduced heat transfer effectiveness, while SHX faults reflect more prominent changes downstream. The ACM faults illustrate relatively moderate temperature deviations due to the air cycle machine’s internal regulation mechanisms, yet these are still discernible from baseline conditions. Ram Air faults, resulting from inlet blockage, manifest pronounced temperature elevations across all sensors, emphasising its critical role as a cooling source. These observations underscore the interdependent behaviour of ECS components and the importance of sensor placement and interpretation in diagnostic evaluations. Legend shows the degradation levels 1 being healthy line where 0.1 indicates 10 percent degradation temperature profile.

Further insights from SESAC simulations [28] reveal that blockage and fouling, despite having distinct physical origins, produce similar effects on heat exchanger performance by reducing effectiveness and thereby increasing temperatures downstream. SESAC simulation results underscore that PHX degradation notably generates a backpressure affecting upstream valves without significantly altering Air Cycle Machine (ACM) operations. Conversely, SHX degradation raises turbine inlet temperatures significantly, increasing turbine work and allowing the compressor to pump more air, demonstrating more straightforward system compensation. These detailed simulation outcomes further illustrate how precise modelling can enhance diagnostic capabilities by delineating component-level effects clearly, providing critical guidance for maintenance interventions.

### 2.2. Theoretical Underpinnings of NDCI

NDCI is computed by combining several key metrics that collectively capture the sensor’s ability to distinguish between normal and faulty operating conditions. Typically, the index is derived from three components:

- Separation Power (SP): For each sensor, SP is calculated by taking the absolute difference between the sensor’s output under fault conditions and its healthy baseline, then normalising this difference by the range of the healthy values. Mathematically, if [yf] is the sensor reading under fault and [yh] is the healthy reading, SP is given by the following:

(1) $$\mathrm{SP}=\frac{\left|y_{f}-y_{h}\;\right|}{range\left(y_{h}\right)+\epsilon }$$

where ε\epsilon ε is a small constant to avoid division by zero.

- Sensitivity (S): Sensitivity captures the relative change in sensor output with respect to deviations in system performance. This is computed by normalising the same absolute difference by the complement of a fault severity indicator (or degradation level), ensuring that even small but significant changes are recognised:

(2) $$S=\frac{\left|y_{f}-y_{h}\;\right|}{\left(1-Deg\right)+\epsilon }$$

where Deg represents the normalised degradation level.

- Uniqueness (U): Uniqueness assesses the degree to which a sensor’s responses differ from those of other sensors. By calculating the average Euclidean distance between the sensor’s fault-response vector and those of its peers, and then normalising by the maximum observed distance, U is defined as follows:

(3) $$U=\frac{avgDistance}{max(avgDistance)}$$

Mathematically, NDCI is typically formulated as the average of these components (NDCI = (SP + S + U)/3), although alternative weighted formulations can be adopted depending on the application requirements. This composite metric thereby captures the multifaceted performance characteristics of a sensor in a single scalar value.

**Figure 3:**
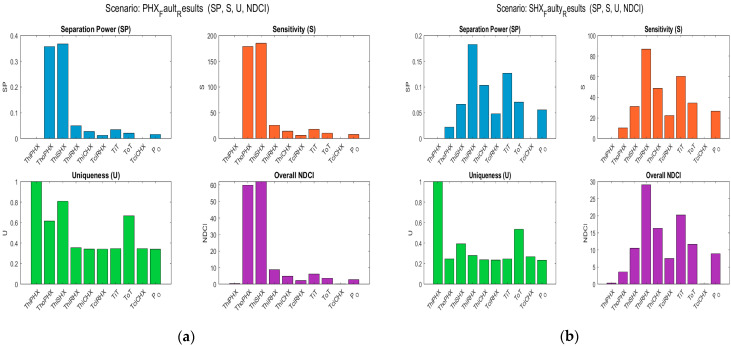

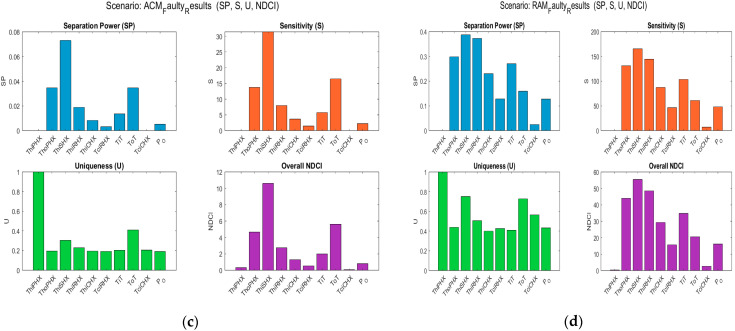
NDCI calculations for ECS fault modes. (**a**) Primary heat exchange fault; (**b**) secondary heat exchange fault; (**c**) Air Cycle Machine fault; (**d**) ram air fault.

Figure 3 presents the computed SP, S, and U values for the four ECS fault modes (PHX, SHX, ACM, and RAM), alongside the resulting NDCI for each sensor. The bar charts highlight which sensors excel in fault separation and sensitivity, as well as how unique each sensor’s response is relative to others. This information is crucial for identifying sensor candidates with high diagnostic relevance.

**Figure 4:**
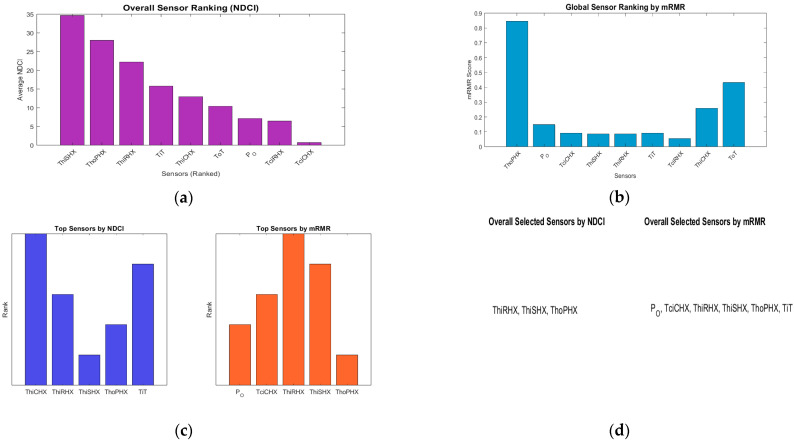
NDCI and mRMR score comparison across fault modes. (**a**) Overall sensor ranking by NDCI; (**b**) global mRMR ranking; (**c**) top sensors comparison by NDCI vs. mRMR; (**d**) minimal sensor subsets comparison.

Figure 4 presents a detailed comparison between sensor rankings derived from NDCI and mRMR methodologies across ECS fault scenarios. Figure 4a illustrates overall sensor rankings based on their average NDCI values, clearly showing sensors like ThiSHX and ThoPHX as highly significant. Conversely, Figure 4b depicts sensor rankings based on mRMR scores. In Figure 4c, a direct comparison highlights discrepancies and overlaps in the top sensors identified by both methods. Sensors such as ThiRHX and ThoPHX consistently rank across both methodologies, underscoring their robust diagnostic value. In contrast, sensors like TiT and P_O show differentiated rankings, indicating their unique contributions are better captured by one criterion over the other.

Figure 4d explicitly addresses the critical issue of minimal sensor subset selection for effective fault detection. Here, subsets identified by each method aim to achieve at least 95% of the maximum diagnostic coverage achievable by utilising all sensors. NDCI-based selection results in a compact subset including three sensors, leveraging sensors with the highest average diagnostic contributions. In contrast, mRMR selects a broader set of six sensors due to its criteria of balancing unique sensor information and redundancy reduction. This comparison highlights that the mRMR criterion inherently seeks broader coverage through diverse information, while NDCI emphasises maximising average diagnostic strength. Thus, utilising both criteria can effectively guide sensor placement and optimisation strategies for robust ECS fault diagnostics.

NDCI and mRMR results derived from the SESAC simulation are shown in Figure 3 and Figure 4. MATLAB-R2024a code for these simulations is available at GitHub [29], facilitating reproducibility and further exploration of the data.

### 2.3. Proposed Methodology for NDCI-MOSOF Integration

The integration of NDCI into MOSOF involves embedding the NDCI within the multi-objective optimisation formulation. In the enhanced framework, NDCI is used as the central diagnostic performance index in the first objective function, which is combined with additional objectives such as cost, reliability (e.g., MTBF or efficiency), and secondary factors (e.g., compatibility, coverage, or benefit-to-cost ratio). For instance, in an OEM scenario, the performance objective is defined as a weighted combination of cost, NDCI, and sensor accuracy. In contrast, in Airlines and MRO scenarios, the performance objective blends NDCI with coverage metrics, while additional objectives capture reliability or efficiency.

**Figure 5:**
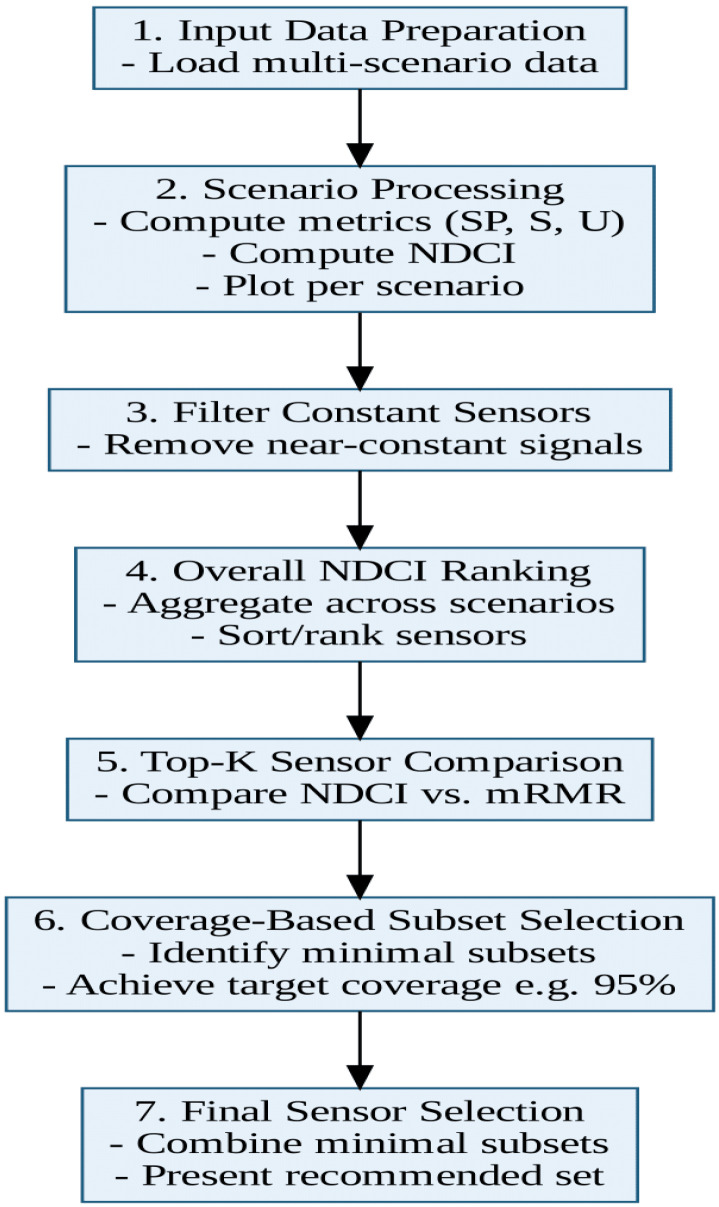
NDCI flowchart.

The flowchart in Figure 5 illustrates the updated procedure for computing NDCI and selecting optimal sensor sets under multiple fault scenarios in MATLAB-R2024a environment. Compared to the earlier version, this refined approach encompasses a multi-scenario perspective, integrates a global mRMR ranking, and incorporates coverage-based subset selection. The following steps summarise the methodology:

1. Input Data Preparation:
  The process begins by loading multi-scenario data from an Excel file (e.g., *SESAC1.xlsx*). Each sheet corresponds to a distinct scenario (or fault type), including a healthy baseline row and multiple fault rows. This step consolidates all necessary information—sensor readings, fault severity indicators (Deg), and baseline values—into a structured format.
2. Scenario Processing (SP, S, U Computation):
  For each scenario, the algorithm calculates three metrics for every sensor:
  - Separation Power (SP): Normalised difference between fault and healthy measurements.
  - Sensitivity (S): Relative change in sensor output scaled by the fault severity.
  - Uniqueness (U): Distinctiveness of a sensor’s fault signatures compared to those of other sensors in the same scenario.
  These metrics are then averaged to produce the scenario-level NDCI for each sensor, providing a quantitative measure of diagnostic performance across multiple fault conditions.
3. Filtering Constant Sensors:
  After processing all scenarios, sensors that exhibit negligible variation (below a specified threshold) are removed to eliminate redundant or uninformative signals. This ensures that subsequent analyses focus only on sensors capable of contributing meaningful diagnostic information.
4. Aggregating Scenario Data for Global Analysis:
  The NDCI values and the underlying fault data from each scenario are compiled into global matrices. This consolidated view facilitates system-wide comparisons and ensures consistency when deriving overall sensor rankings.
5. Overall NDCI Ranking:
  An overall average NDCI is computed across all scenarios, and sensors are ranked accordingly. This ranking highlights those sensors that consistently demonstrate high diagnostic value in multiple fault conditions.
6. Coverage-Based Subset Selection (Per Scenario):
  Each scenario undergoes a coverage analysis, which measures how effectively a subset of sensors (chosen in descending order of their NDCI or mRMR scores) captures the scenario’s total diagnostic capability. Once the subset meets a predefined coverage threshold (e.g., 95% of maximum possible NDCI), no further sensors are added.
7. Union of Minimal Subsets:
  The minimal subsets identified per scenario are combined to form a single, system-wide sensor set. This ensures that unique fault modes in different scenarios are adequately covered without inflating sensor counts unnecessarily.
8. Final Sensor Selection and Output:
  The resulting sensor set, along with the intermediate rankings (NDCI-based and mRMR-based) and coverage analyses, is presented as the final output. This includes visualisations comparing top-k sensors by NDCI versus mRMR and any additional metrics relevant to cost or reliability.

By adopting this flow, the approach ensures robust sensor selection across multiple fault scenarios, balances redundancy with coverage, and incorporates NDCI insights. The methodology thereby offers a comprehensive framework that can be adapted to diverse domains, from aerospace to industrial process monitoring.

**Figure 6:**
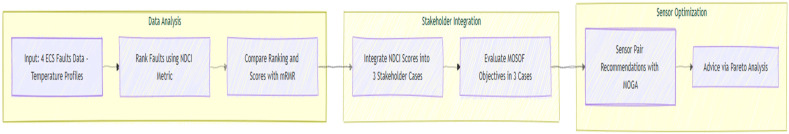
Research methodology.

This methodology is operationalised via a MOGA that selects sensor pairs meeting specific constraints (e.g., budget limits and minimum performance thresholds). The optimisation process generates a Pareto front that represents trade-offs among the objectives. Detailed simulation studies demonstrate that this NDCI-MOSOF integration not only broadens the feasible solution space but also yields sensor configurations that are more attuned to application-specific performance needs. The research methodology is presented in Figure 6.

Grounding sensor evaluation in NDCI enables a more precise and application-oriented sensor selection process, thus paving the way for future research in domain-specific sensor optimisation strategies.

## 3. Multi-Objective Optimisation Framework

The sensor selection problem is formulated as a multi-objective optimisation task where a binary decision vector represents the inclusion or exclusion of each sensor in the network. In the formulation, each sensor in the pool is associated with a set of performance attributes—most notably, the NDCI, along with secondary criteria such as accuracy, cost, reliability (or efficiency), and compatibility (or coverage). The objective is to identify sensor pairs that yield optimal trade-offs among these metrics while satisfying system-level constraints.

Table 1 offers a focused, NDCI-centric perspective on how the three primary aviation stakeholders, OEM, Airlines, and MRO, integrate diagnostic performance into their operational objectives. By placing NDCI at the forefront of each decision-making criterion, the table captures both the high-level trade-offs and specific considerations (e.g., cost, reliability, and downtime) that influence sensor optimisation strategies. This view not only underscores the importance of aligning sensor choices with each stakeholder’s priorities but also clarifies how a unified performance metric, such as NDCI, can streamline communication and goal setting across the aircraft’s entire lifecycle.

### 3.1. Problem Formulation and Decision Variables

Let **x** = [x_1_, x_2_, …, x_n_] ᵀ be the binary decision vector, where n is the total number of sensors available. Each element x_i_ ∈ {0, 1} indicates whether sensor i is selected (x_i_ = 1) or not (x_i_ = 0). A key constraint requires that exactly two sensors must be chosen, which is expressed mathematically as follows: Σ (from i = 1 to n) x_i_ = 2.

This binary formulation allows a direct representation of sensor configurations and enables the use of evolutionary algorithms for optimisation.

### 3.2. Objective Functions

The framework defines four objectives that together capture the multifaceted performance requirements. These objectives are combined into a vector-valued function, where F(x) = [f_1_(x), f_2_(x), f_3_(x), f_4_(x)].

- Performance (f_1_):
  This objective is a weighted combination of normalised NDCI and an additional performance metric (for example, accuracy in the OEM scenario or a blend of normalised NDCI and coverage for Airlines and MRO). It is expressed as follows:

f_1_(x) = −[w_1_ · (Σ x_i_ · NDCI_i_/max (NDCI)) + w_2_ · (Σ x_i_ · p_i_/max(p))], (4)

where p_i_ represents the secondary performance metric (such as accuracy), w_1_ and w_2_ are weights with w_1_ + w_2_ = 1, and the negative sign indicates that the objective is minimised.

- Cost (f_2_):

The cost objective is defined as the total cost of the selected sensors:

f_2_(x) = Σ (x_i_ · c_i_),

where c_i_ is the cost of sensor i.

- Reliability/Efficiency (f_3_):
  This objective promotes robust sensor performance by being defined as the negative of a normalised reliability (or efficiency) measure:

f_3_(x) = −(Σ x_i_ · r_i_/max(r)),

where r_i_ denotes the reliability (e.g., mean time between failures) or efficiency of sensor i.

- Secondary Criterion (f_4_):
  For OEM applications, this is typically the negative of normalised compatibility; for Airlines and MRO, a benefit-to-cost ratio is adopted. For example, in the OEM scenario:

f_4_(x) = −(Σ x_i_ · d_i_/max(d)),

where d_i_ represents the compatibility of sensor i. In other scenarios, f_4_ may be defined as follows:

f_4_(x) = −(Σ x_i_ · q_i_)/(Σ x_i_ · c_i_),

with q_i_ being a composite benefit measure (such as the sum of normalised NDCI, coverage, and efficiency).

These four objectives collectively express the trade-offs among diagnostic performance, cost, reliability (or efficiency), and secondary considerations.

### 3.3. Optimisation Setup

The optimisation problem is subject to several constraints that ensure system viability:

- Binary Selection Constraint:
  ○ Each sensor decision variable must be binary, i.e., x_i_ ∈ {0, 1} for all i = 1, …, n, with Σ (from i = 1 to n) x_i_ = 2.
- Budget Constraint:
  ○ The total cost of the selected sensors must not exceed a predefined budget B:
    Σ (x_i_ · c_i_) ≤ B.
- Performance Thresholds:
  Minimum acceptable levels are imposed on key metrics:
  - The sum of NDCI values must be at least Tₙdc_i_:
    Σ (x_i_ · NDCI_i_) ≥ Tₙdc_i_.
  - The average accuracy (or equivalent performance metric) must be at least Aₘ_i_ₙ:
    (Σ (x_i_ · p_i_))/(Σ x_i_) ≥ Aₘ_i_ₙ.
  - The total reliability (or efficiency) must be at least Rₘ_i_ₙ:
  Σ (x_i_ · r_i_) ≥ Rₘ_i_ₙ.

A MOGA is employed to solve this optimisation problem. MATLAB-R2024a’s “gamultiobj” function is used with a custom creation function that ensures every candidate solution in the initial population consists of exactly two selected sensors. Custom mutation and crossover functions are designed to maintain feasibility (i.e., preserving the constraint Σ x_i_ = 2) throughout the evolutionary process. The algorithm iteratively evolves the population until convergence is reached, resulting in a Pareto front of non-dominated solutions that represent the best trade-offs among the objectives.

The resulting Pareto set provides a diverse array of sensor-pair configurations. For visualisation, pairwise scatter plots are generated for every combination of two objectives. Each plotted point represents a sensor pair and is annotated with the corresponding sensor names. This comprehensive analysis supports informed decision-making in complex sensor network implementations and demonstrates the benefits of integrating NDCI into the MOSOF.

## 4. Experimental Results and Analysis

### 4.1. Simulation Setup and Parameter Specifications

The proposed NDCI-MOSOF integration was evaluated using simulation studies for three representative scenarios: OEM, Airlines, and MRO. For each case, the sensor pool comprised nine sensors with ECS simulation names and NDCI values. In the OEM scenario, fixed values were used for key parameters (e.g., cost, accuracy, MTBF, and compatibility). In contrast, for the Airlines and MRO cases, the additional attributes (cost, coverage, reliability, or efficiency) were generated randomly within realistic bounds to induce nontrivial trade-offs. For instance, in the Airlines scenario, cost was varied between 240 and 280 units, coverage ranged from 0.90 to 0.98, and reliability was randomised between 5100 and 5700. Similar ranges were used for the MRO case, with efficiency values sampled uniformly between 0.80 and 0.95.

MOGA was configured with a population size of 200, a maximum of 500 generations, and a Pareto fraction of 35%. A custom creation function ensured that every individual in the initial population corresponded to exactly two sensors. Other GA parameters, such as mutation (using a feasible-adaptive strategy) and intermediate crossover, were tuned to maintain diversity and feasibility of solutions. Constraints were imposed on total cost, minimum sum of NDCI values, average performance (e.g., accuracy or coverage), and aggregated reliability/efficiency. These constraints were slightly relaxed to ensure a rich solution space, with several sensor pairs meeting all criteria.

In addition to evaluating our proposed method, we conducted comparative studies contrasting the performance of the NDCI-MOSOF against conventional approaches such as mRMR. The analysis, supported by quantitative metrics, including diagnostic accuracy and cost efficiency, demonstrates that our integration not only reduces sensor count but also achieves superior trade-offs among key performance objectives.

### 4.2. Pareto Front Analysis and Sensor Pair Configurations

The optimisation produced a diverse Pareto front capturing the trade-offs among the four objectives: performance (a combination of normalised NDCI and a secondary metric), cost, reliability/efficiency, and a secondary benefit-to-cost or compatibility measure. Detailed pairwise scatter plots (six subplots for every combination of two objectives) reveal that sensor pair solutions span a wide range of trade-offs.

**Figure 7:**
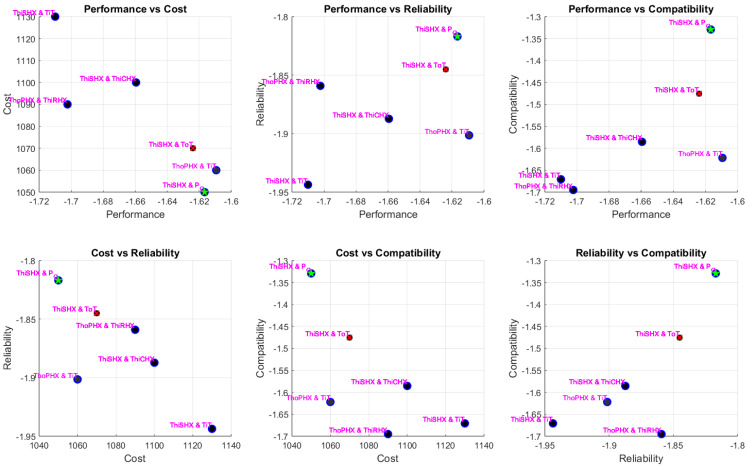
OEM case pairwise objective scatter plots.

**Figure 8:**
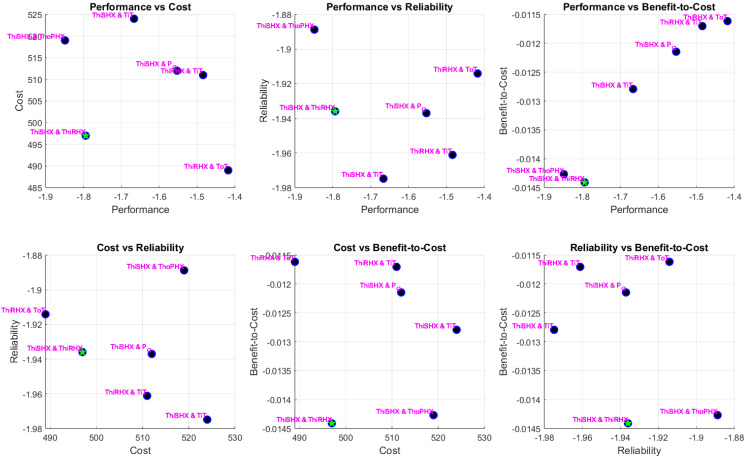
Airlines case pairwise objective scatter plots.

**Figure 9:**
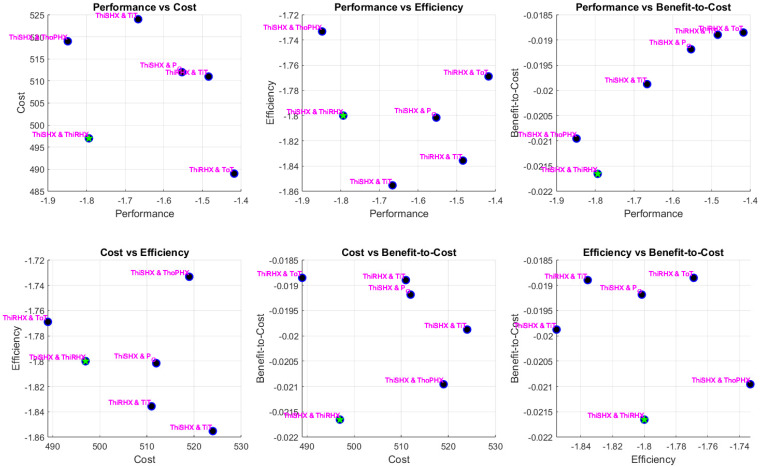
MRO case pairwise objective scatter plots.

Simulation results of each scenario are presented below in Figure 7, Figure 8 and Figure 9. The MATLAB-R2024a code of the simulations is available at GitHub [30], facilitating reproducibility and further exploration of the data. Each point in these plots represents a sensor pair, annotated with sensor names. In the OEM scenario, for example, sensor pairs such as “ThiSHX (Temperature, hot, inlet Secondary Heat Exchanger)–TiT (Temperature, inlet Turbine)” and “ThiRHX (Temperature, hot, inlet Reheater)–P_O (Pack Outlet)” emerged as promising candidates. Feasible solutions (those that met all constraints) are clearly distinguishable from infeasible ones, with blue markers denoting feasible sensor pairs and red crosses indicating violations. The optimal pair (selected as the first feasible solution with the best trade-off among objectives) is highlighted with a green star. Notably, the incorporation of NDCI as a central performance metric enabled greater discrimination between sensor pairs, revealing non-obvious combinations that offer balanced performance and cost advantages.

## 5. Discussion

The experimental results demonstrate that integrating the NDCI into the MOSOF enhances sensor selection in diagnostic applications for complex systems by integrating multiple aspects and different users’ objectives into one place for sensor design implementations. In contrast to the conventional MOSOF, the NDCI-MOSOF approach introduces a performance metric that captures subtle yet critical differences among sensor outputs. This improved discrimination is evident in the broader Pareto spread and more diversified sensor pair configurations obtained in the simulations.

One major implication is that the NDCI-centric framework facilitates a more balanced trade-off between performance and cost. By incorporating NDCI as a core metric in the performance objective, the framework successfully identifies sensor pairs that are not only cost-effective but also exhibit superior sensitivity and fault detection capabilities. This is particularly relevant in applications such as control systems, process monitoring, and security, where high-fidelity sensor performance is essential.

Furthermore, the adaptability of the proposed method to different application scenarios (OEM, Airlines, MRO) underscores its versatility. The random scattering of secondary attributes in the Airlines and MRO cases further validates the framework’s robustness against non-ideal correlations among sensor attributes. The ability to adjust constraints and objective weightings makes the framework suitable for a wide range of sensor implementations, ensuring that system-specific requirements are met.

The experimental results confirm the efficacy of the NDCI-MOSOF integration; however, practical deployment in real-world environments involves challenges such as computational complexity and sensor calibration under dynamic conditions. Future work will address these challenges by exploring modular implementation strategies and real-time data integration, thereby enhancing the scalability of our approach across large-scale aircraft sensor networks.

However, this study also highlights potential limitations, such as the reliance on simulation data. Future research should focus on validating the NDCI-MOSOF approach with empirical data. Additionally, extending the framework to incorporate dynamic sensor behaviour and real-time adaptation remains a promising research direction.

## 6. Conclusions

This study presents a novel integration of the NDCI into the MOSOF to address the nuanced performance requirements of complex sensor systems. By embedding NDCI as a central performance metric and augmenting the framework with additional objectives (such as cost, reliability/efficiency, and secondary factors like compatibility or benefit-to-cost ratio), the proposed NDCI-MOSOF approach provides a more precise and application-specific sensor selection process.

Simulation studies across OEM, Airlines, and MRO scenarios demonstrate that the enhanced framework can generate a diversified Pareto front of feasible sensor pair configurations. These results indicate improved discrimination among sensor pairs, leading to a better balance between performance and economic considerations based on the stakeholders/user requirements. The approach also shows strong potential for adaptation to other complex system diagnostic applications.

In conclusion, integrating NDCI into MOSOF represents a significant advancement in sensor optimisation methodologies. The enhanced framework not only refines sensor selection by focusing on critical performance attributes but also offers a flexible tool for tailoring sensor configurations to diverse and dynamic application environments. Future work will focus on real-world validation and dynamic adaptation to further extend the applicability of the proposed method.

## Figures and Tables

**Table 1:** Sensor application domains comparison.

Main Application Areas	Key Functions	Key Benefits	Examples	MOSOF: Sensor Selection	MOSOF: Sensor Placement	MOSOF: Data Processing	MOSOF: Sensor Operation
Diagnostics	Fault detection, predictive maintenance, calibration	Early fault identification, reduced downtime, system accuracy	Detecting machine wear, diagnosing system errors	Accuracy for fault detection, sensitivity to anomalies	Strategic positioning for fault-prone areas	Fault trend analysis, anomaly detection algorithms	Periodic calibration, low power consumption
Control	Automation, energy management, optimisation	Real-time adjustments, resource savings, enhanced performance	Optimising HVAC systems, adjusting power usage dynamically	Sensors capable of real-time adjustments	Placement for real-time feedback integration	Real-time control feedback loops, optimisation algorithms	Seamless integration with control systems
Monitoring and Reporting	Environmental compliance, data logging, user interfaces	Regulatory adherence, historical insights, user interaction	Logging air quality data, monitoring production trends	Sensors with wide environmental parameter ranges	Distributed placement for comprehensive monitoring	Data aggregation, compliance reports, and visualisation	Reliable operation under varying conditions
Safety and Security	Surveillance, access control, safety assurance	Improved security, system integrity, risk reduction	Securing restricted areas, detecting unauthorised entry	High-security sensors, tamper-proof capabilities	Coverage for all critical and high-risk areas	Event driven processing, security breach detection	Resilient operation in high-security environments
System and Process Optimisation	Efficiency improvement, operational reliability	Cost savings, process efficiency, stable operations	Streamlining production lines, reducing energy wastage	Energy- efficient, long-lasting, and multi-functional sensors	Optimised placement for process and energy efficiency	Efficiency metrics, operational insights, optimisation models	Sustained performance under demanding conditions

## Data Availability

Data are available in references.

## References

[1] Vachtsevanos G., Lewis F., Roemer M., Hess A., Wu B. (2007). Intelligent Fault Diagnosis and Prognosis for Engineering Systems.

[2] Lei Y., Li N., Guo L., Li N., Yan T., Lin J. (2018). Machinery health prognostics: A systematic review from data acquisition to RUL prediction. Mech. Syst. Signal Process..

[3] Peng H., Long F., Ding C. (2005). Feature selection based on mutual information: Criteria of Max-Dependency, Max-Relevance, and Min-Redundancy. IEEE Trans. Pattern Anal. Mach. Intell..

[4] Aljawarneh M., Hamdaoui R., Zouinkhi A., Boussaid B., Abdelkrim M.N. Energy efficiency approaches in Wireless Sensor Networks. Proceedings of the 2022 IEEE 21st International Conference on Sciences and Techniques of Automatic Control and Computer Engineering, STA 2022-Proceedings.

[5] Jo I., Lee S., Oh S. (2019). Improved measures of redundancy and relevance for mRMR feature selection. Computers.

[6] Santi L.M., Sowers T.S., Aguilar R.B. Optimal sensor selection for health monitoring systems. Proceedings of the 41st AIAA/ASME/SAE/ASEE Joint Propulsion Conference and Exhibit.

[7] Yang S.-M., Qiu J., Liu G.-J., Yang P. (2013). Sensor Selection and Optimization for Aerospace System Health Management under Uncertainty Testing. Trans. Jpn. Soc. Aeronaut. Space Sci..

[8] Esperon-Miguez M., Jennions I.K., Escobar I.C., Hanov N. (2019). Simulating faults in a boeing 737-200 environmental control system using a thermodynamic model. Int. J. Progn. Health Manag..

[9] Jia L., Ezhilarasu C.M., Jennions I.K. (2023). Cross-Condition Fault Diagnosis of an Aircraft Environmental Control System (ECS) by Transfer Learning. Appl. Sci..

[10] Xu Z., Guo Y., Saleh J.H. (2022). Multi-objective optimization for sensor placement: An integrated combinatorial approach with reduced order model and Gaussian process. Measurement.

[11] Lee K., Han S., Pham V.H., Cho S., Choi H.-J., Lee J., Noh I., Lee S.W. (2021). Multi-objective instance weighting-based deep transfer learning network for intelligent fault diagnosis. Appl. Sci..

[12] Kabashkin I., Perekrestov V., Tyncherov T., Shoshin L., Susanin V. (2024). Framework for Integration of Health Monitoring Systems in Life Cycle Management for Aviation Sustainability and Cost Efficiency. Sustainability.

[13] Suslu B., Ali F., Jennions I.K. (2023). Understanding the Role of Sensor Optimisation in Complex Systems. Sensors.

[14] Xu J., Wang Y., Xu L. (2015). PHM-Oriented Sensor Optimization Selection Based on Multiobjective Model for Aircraft Engines. IEEE Sensors J..

[15] Farschman C.A., Viswanath K.P., Ydstie B.E. (1998). Process systems and inventory control. AIChE J..

[16] Clark E., Brunton S.L., Kutz J.N. (2021). Multi-Fidelity Sensor Selection: Greedy Algorithms to Place Cheap and Expensive Sensors with Cost Constraints. IEEE Sens. J..

[17] Civera M., Pecorelli M.L., Ceravolo R., Surace C., Fragonara L.Z. (2021). A multi-objective genetic algorithm strategy for robust optimal sensor placement. Comput.-Aided Civ. Infrastruct. Eng..

[18] Hare J., Gupta S., Najjar N., D’Orlando P., Walthall R. System-Level Fault Diagnosis with Application to the Environmental Control System of an Aircraft. Proceedings of the SAE 2015 AeroTech Congress & Exhibition.

[19] de Souza Mello F.M., Gomes G.F. (2025). Sensor placement optimization for composite aircraft structures: A multi-objective Kriging-based approach. Compos. Struct..

[20] New Aircraft Cabin Environment Sensor Certified for Boeing 737-Avionics International. https://www.aviationtoday.com/2021/03/23/new-aircraft-cabin-environment-sensor-certified-boeing-737/.

[21] Ezhilarasu C.M., Skaf Z., Jennions I.K. (2021). A Generalised Methodology for the Diagnosis of Aircraft Systems. IEEE Access.

[22] Mao L., Davies B., Jackson L. (2017). Application of the Sensor Selection Approach in Polymer Electrolyte Membrane Fuel Cell Prognostics and Health Management. Energies.

[23] Lee D., Lee I., Kim Y., Joo S.C., Choi J.H. (2024). Sensor set optimization by functional model and Bayesian network for fault diagnosis of turbine generator lubrication system. Eng. Appl. Artif. Intell..

[24] Planès T., Delbecq S., Pommier-Budinger V., Bénard E. (2023). Modeling and Design Optimization of an Electric Environmental Control System for Commercial Passenger Aircraft. Aerospace.

[25] Liu Q., Zhuang L., Wen J., Dong B., Liu Z. (2022). Thermodynamic Optimization of Aircraft Environmental Control System Using Modified Genetic Algorithm. Processes.

[26] House J.M., Seem J.E. (2012). Systems and Methods for Fault Detection of Air Handling Units.

[27] Shao Q., Kumagai G., Dong B., Kim K., Singhai M. (2020). Pattern Classification System with Smart Data Collection for Environmental Control System Fault Isolation.

[28] Jennions I., Ali F. (2021). Assessment of heat exchanger degradation in a boeing 737-800 environmental control system. J. Therm. Sci. Eng. Appl..

[29] B.Suslu-NDCI_Calculations. https://github.com/ssl8/ECS-sensor-optimisation/blob/4174e20aff3e1c189d72aa9c75fbd104a5c339c7/multiFaultComparison.m.

[30] B.Suslu-MOSOF_ECS_Case_Outlines. https://github.com/ssl8/ECS-sensor-optimisation/blob/0ce6ca4df91cb1fc3f2c98b2483d4b9b6caed0d9/NDCI_MOSOF_ECS.m.

